# Vascular endothelial growth factor-D over-expressing tumor cells induce differential effects on uterine vasculature in a mouse model of endometrial cancer

**DOI:** 10.1186/1477-7827-8-84

**Published:** 2010-07-08

**Authors:** Jane E Girling, Jacqueline F Donoghue, Fiona L Lederman, Leonie M Cann, Marc G Achen, Steven A Stacker, Peter AW Rogers

**Affiliations:** 1 Centre for Women's Health Research, Monash Institute of Medical Research and Monash University Department of Obstetrics and Gynaecology, Monash Medical Centre, 246 Clayton Rd, Clayton, VIC 3168, Australia; 2 Ludwig Institute for Cancer Research, PO Box 2008, Royal Melbourne Hospital, Victoria, Australia

## Abstract

**Background:**

It has been hypothesised that increased VEGF-D expression may be an independent prognostic factor for endometrial cancer progression and lymph node metastasis; however, the mechanism by which VEGF-D may promote disease progression in women with endometrial cancer has not been investigated. Our aim was to describe the distribution of lymphatic vessels in mouse uterus and to examine the effect of VEGF-D over-expression on these vessels in a model of endometrial cancer. We hypothesised that VEGF-D over-expression would stimulate growth of new lymphatic vessels into the endometrium, thereby contributing to cancer progression.

**Methods:**

We initially described the distribution of lymphatic vessels (Lyve-1, podoplanin, VEGFR-3) and VEGF-D expression in the mouse uterus during the estrous cycle, early pregnancy and in response to estradiol-17beta and progesterone using immunohistochemistry. We also examined the effects of VEGF-D over-expression on uterine vasculature by inoculating uterine horns in NOD SCID mice with control or VEGF-D-expressing 293EBNA tumor cells.

**Results:**

Lymphatic vessels positive for the lymphatic endothelial cell markers Lyve-1, podoplanin and VEGFR-3 profiles were largely restricted to the connective tissue between the myometrial circular and longitudinal muscle layers; very few lymphatic vessel profiles were observed in the endometrium. VEGF-D immunostaining was present in all uterine compartments (epithelium, stroma, myometrium), although expression was generally low. VEGF-D immunoexpression was slightly but significantly higher in estrus relative to diestrus; and in estradiol-17beta treated mice relative to vehicle or progesterone treated mice. The presence of VEGF-D over-expressing tumor cells did not induce endometrial lymphangiogenesis, although changes were observed in existing vessel profiles. For myometrial lymphatic and endometrial blood vessels, the percentage of profiles containing proliferating endothelial cells, and the cross sectional area of vessel profiles were significantly increased in response to VEGF-D in comparison to control tumor cells. In contrast, no significant changes were noted in myometrial blood vessels. In addition, examples of invading cells or tumor emboli were observed in mice receiving VEGF-D expressing 293EBNA cells.

**Conclusions:**

These results illustrate that VEGF-D over-expression has differential effects on the uterine vasculature. These effects may facilitate VEGF-D's ability to promote endometrial cancer metastasis and disease progression.

## Background

To date, minimal research has been directed at elucidating the mechanisms responsible for normal and abnormal growth and development of the endometrial lymphatic vasculature [1-3]. This is despite the hypothesised or known role for this vascular system in various gynaecological pathologies, including endometrial cancer. We recently used a specific marker of lymphatic endothelial cells (podoplanin [D2-40]) to describe the distribution of lymphatic vessels within the human uterus [4]. Lymphatic vessels were observed in both the myometrium and endometrium, with fewer vessels present in the endometrial functionalis compared to the basalis. In endometrial adenocarcinoma, significant increases in vessel density were observed in the peri-tumoral relative to normal basalis and myometrium. Vascular space invasion was also observed, with the vessels affected exhibiting a mixed lymphatic and blood endothelial cell phenotype [4]. In other studies of endometrial cancer, increased peri-tumoral lymphatic vessel density was a marker of higher grade endometrial tumours with a less favourable prognosis [5,6]. The presence of vascular space invasion has also been reported to be a strong predictor of lymph node metastasis, disease recurrence and poor prognosis [7-10]. In combination, these studies highlight the importance of the uterine lymphatic vasculature to endometrial cancer progression. However, the specific features of endometrial tumour cells that promote this dissemination are not well understood.

A growth factor involved in both angiogenesis and lymphangiogenesis is vascular endothelial growth factor (VEGF)-D. VEGF-D, and the related protein VEGF-C, are initially produced as full-length forms, which can be enzymatically cleaved to generate smaller polypeptides or isoforms with enhanced receptor binding affinities [11-19]; various isoforms of both growth factors are present within the human endometrium [4]. In humans, the mature and fully processed forms of VEGF-C and VEGF-D bind and activate VEGF receptor-2 (VEGFR-2) and VEGFR-3, which are found predominately on blood and lymphatic endothelial cells, respectively [20]. {Note: Mouse VEGF-D does not interact with mouse VEGFR-2. [21]} VEGF-D deficient mice lack an overt phenotype and have normal vasculature and fertility, suggesting that embryonic lymphangiogenesis is not dependent on VEGF-D [22,23]. However, several studies have shown that VEGF-D stimulates lymphangiogenesis and/or angiogenesis *in vivo *in the adult [24,25].

Increased VEGF-D expression has been observed in several reproductive tract cancers in association with lymph node metastases [26]. In endometrial adenocarcinoma, increased VEGF-D protein expression was observed in tumour relative to normal cycling endometrium [4]. Increased VEGF-D expression has also been reported to be an independent prognostic factor for endometrial cancer progression and may predict myometrial invasion and lymph node metastasis [27].

Previous studies have demonstrated a tissue-specific effect of VEGF-D on vascular remodeling. In this study, our aim was to determine the response of uterine vasculature to VEGF-D expressing cells in a mouse model of endometrial cancer. In the first instance, we described the distribution of lymphatic vessels in the mouse uterus using the lymphatic endothelial cell markers lymphatic endothelial hyaluronan receptor 1 (Lyve-1), podoplanin and VEGF receptor-3 (VEGFR-3). Lymphatic vessels positive for the above markers were largely restricted to the myometrium with few vessels present in the endometrium. We hypothesised that VEGF-D over-expression would stimulate growth of new lymphatic vessels into the endometrium. Interestingly, endometrial lymphangiogenesis did not occur in response to VEGF-D over-expression; however, enlargement and proliferation of existing endometrial blood and myometrial lymphatic vessels was observed. In addition, tumour cells were also observed within lymphatic vessel profiles.

## Methods

### Animals

All mice were housed under controlled environmental conditions (20°C, 12 h light per day) and provided with food and water ad libitum. Animal studies were approved by the Monash Medical Centre Animal Ethics Committee A.

### Distribution of lymphatic vessels and VEGF-D expression in the mouse uterus

Uterine tissues were collected from C57BL/6J × CBA mice (7-13 weeks, 18-28 g, Monash Animal Services, Monash University, Victoria, Australia) at different stages of the estrous cycle (diestrus, proestrus and estrus; n = 7-9), during early pregnancy (days 1-4, n = 4-5) and following treatment with both exogenous estradiol-17β and progesterone (as per published protocols [28-31]. Anaesthetized mice were bilaterally ovariectomized and left for 7 days following ovariectomy. One group of mice was treated with a single s.c. injection of estradiol-17β (100 ng in 100 μl peanut oil), followed by a no-treatment day and three consecutive daily s.c. injections of progesterone (1 mg in 100 μl peanut oil), before dissection 24 h after the final hormone injection (n = 8) [28,31]. Other groups were injected with the vehicle (100 μl peanut oil, n = 7), or progesterone injections only (n = 7). A final group of ovariectomised mice received a single s.c. injection of estradiol-17β (100 ng in 100 μl peanut oil) and was dissected 24 h later (n = 7) [29,30]). All uterine tissues were removed and immersion fixed in 10% buffered formalin for 2 h before processing prior to immunohistochemistry for blood vessels, lymph vessels and VEGF-D immunoreactivity.

### VEGF-D overexpression in the mouse uterus

#### Preparation of tumor cells

Two cell lines were used: human embryonic kidney 293EBNA (Epstein-Barr virus nuclear antigen) and 293EBNA-VEGF-D cells. The latter cell line is stably transfected with an expression construct encoding full-length human VEGF-D Flag tagged at the C-terminus, as previously described [19], and has been shown to induce lymphangiogenesis in another model [25]. 293EBNA cells were chosen for the current study as they are a well characterized cell line that does not express detectable levels of VEGF-A, VEGF-C or VEGF-D [25]. This allows the specific effects of VEGF-D to be investigated without the confounding effects of other VEGF family members. Both cell lines were maintained in culture (DMEM/F12, Invitrogen; Melbourne, Australia) with 10% fetal calf serum (CSL; Melbourne, Australia), antibiotics and glutamine (Invitrogen; Melbourne, Australia) under normoxic conditions (5% CO_2 _and air). The VEGF-D-expressing cells were cultured in the presence of hygromycin B (100 μg/ml: Invitrogen) as the VEGF-D expression vector encodes the resistance gene for this antibiotic.

#### Uterine inoculation with tumor cells

Uteri from normal cycling NOD/SCID (Non-Obese Diabetic/Severe Combined Immunodeficiency) mice were inoculated with tumor cells following a procedure adapted from Hashii *et al*. [32]. One group of animals received 293EBNA cells (n = 9) and a second group received 293EBNA-VEGF-D cells (n = 9). Following anaesthesia with Ketamine and Xylazine (100 mg/kg and 5 mg/kg respectively), a 5% agar plug was inserted into the vagina of each mouse to prevent leakage of injected cells. The upper region of a uterine horn was then exteriorized through a small incision in the lower ventral region, just below the kidney. Before the injection, surgical thread (Coated Vicryl, Johnson and Johnson, Australia) was placed around the upper region of the horn near the oviduct. A needle was inserted into the upper region of the uterine horn and the inner region of the horn then damaged by scraping with a 25 gauge needle. A syringe containing 5 × 10^6 ^cells in 50 μl media was attached to the needle and the cells injected. The surgical thread was then tied off to prevent backflow of the injected cells. The second uterine horn received an intramuscular injection of a similar cell mix. A 25 gauge needle was inserted along the outer muscular wall of the upper region of the uterus near the oviduct. The injection site was then ligatured to prevent backflow.

NOD SCID mice were left for period of 4 weeks to allow tumors to form. After 4 weeks, the uterine tissue was collected from each animal and formalin fixed for histological examination. Each horn was separated into an upper region (including the ovary, ligature and upper uterine horn; including injection site) and a lower distal region (including the lower uterine horn and cervical junction; away from injection site). Uterine samples were orientated longitudinally and blocked in paraffin.

### Immunohistochemistry

Antibodies against Lyve-1 (polyclonal goat anti-human Lyve-1, 1 μg/ml; #AF2089, R&D, Minneapolis, MN, USA), podoplanin (goat anti-mouse podoplanin, 1 μg/ml, #AF3244, R&D) and VEGFR-3 (monoclonal rat anti VEGFR-3, 2 μg/ml; clone AFL4, now commercially available from R&D Systems) were used to identify lymphatic vessels. Blood vessels were identified using CD31 (rat anti mouse CD31, 5 μg/ml; #553370, BD Pharmingen, Franklin Lakes, NJ, USA). When required, dual immunostaining was used to illustrate blood (CD31) versus lymphatic (podoplanin) vessel distribution and to identify proliferating cells (monoclonal mouse anti-proliferating cell nuclei antibody {PCNA}, 1 μg/ml, NCL-PCNA, Novacastra Laboratories, Newcastle, United Kingdom) in lymphatic or blood vessels. Mouse VEGF-D was detected using a polyclonal goat anti-mouse VEGF-D antibody (1 μg/ml, #AF469, R&D). An antibody directed specifically against human VEGF-D (monoclonal mouse anti-human VEGF-D, 5 μg/ml, #MAB286, R&D) was used to identify 293EBNA cells transfected with VEGF-D within mouse tissues. An antibody directed against human mitochondrial antigen was used to identify non-transfected 293EBNA cells (monoclonal mouse anti-human mitochondria, 250 μg/ml, MAB1273, Chemicon, Temecula, CA, USA; does not cross react with mouse antigens).

Following dewaxing and rehydration, antigen retrieval was performed by incubating sections (3 or 5 μm) in 0.1% (1 mg/ml) pepsin in 3% acetic acid (10 min, 37°C) (for CD31 staining) or by microwaving sections in tri-sodium citrate buffer (for all other antibodies) (10 nM Na-Citrate, pH6). For all protocols except VEGFR-3, sections were then incubated with H_2_O_2 _(3% dH_2_O_2_, 47% H_2_O, 50% methanol, 10 min) to quench endogenous peroxidase and protein blocking solution (PBA, Immunon Shandon, PA, USA) to prevent non-specific binding. For immunostaining of VEGFR-3, the endogenous peroxidase was quenched using 0.2% NaN_3_/0.6% H_2_O_2 _in methanol (30 min) followed by PBA. Following incubation with primary antibodies (1 h at 37°c or overnight at 4°C), a biotinylated secondary antibody was applied (Lyve-1: ready to use biotinylated rabbit anti-goat IgG, 15 min, #50-232Z, Zymed, San Francisco, CA, USA, VEGFR-3 and CD31: biotinylated goat anti-rat IgG, 1:200 for 1 hour, #AP183B, Chemicon; podoplanin: biotinylated rabbit anti-goat IgG, 30 min, A10518, Invitrogen, Carlsbad, CA, USA, mouse VEGF-D: biotinylated rabbit anti-goat, 1:200 for 30 min, #81-1644, Zymed; human VEGF-D: mouse on Mouse (MOM) kit, #BMK-2202, Vector Laboratories, Burlingame, USA, human cells: LSAB2 System-HRP, DakoCytomation, Carpinteria, CA, USA; PCNA: EnVision+ System-HRP (AEC), DakoCytomation).

Immunostaining for Lyve-1, VEGFR-3, VEGF-C, mouse VEGF-D and human mitochondrial antigen was visualized after incubation in LSAB2 streptavidin-HRP (DakoCytomation) for 15 min followed by the chromogen 3,30-diaminobenzidine (DAB) (Sigma-Aldrich). Immunostaining for CD31 and Lyve-1 (note that two different protocols were used for Lyve-1 immunostaining making use of DAB or vector blue as appropriate) was visualized after incubation with LSAB2 streptavidin-AP (15 min, DakoCytomation) followed by vector blue chromogen (10 min, Vector Laboratories, CA, USA). Immunostaining for podoplanin was visualized after incubation with the Vectastain ABC kit (Vector Labs Inc, Burlingame, CA) for 10 min followed by DAB. Immunostaining for human VEGF-D was performed using the mouse on mouse (MOM) kit according to manufacturer's instructions (VECTOR Laboratories, Burlingame, USA) and was visualized with DAB (Sigma-Aldrich).

When dual immunostaining was used to detect CD31 and podoplanin, the CD31 protocol outlined above was conducted first, followed by the podoplanin protocol. When dual immunostaining for PCNA and either CD31 or Lyve-1, immunostaining for the endothelial cell-specific antibody was conducted first, followed by immunostaining with the primary antibody against PCNA. PCNA immunostaining was visualized with Envision (Dakocytomation) followed by aminoethyl carbazole (AEC) (Zymed, CA, USA).

For each protocol, a negative isotype matched control was prepared by replacing the primary antibodies with the appropriate matched IgG at the same concentrations as those of the primary antibodies.

### Image analysis

All image analysis was performed using the Analytical Imaging System (AIS 30, Rev 1.7; Imaging Research Inc., GE Healthcare Bio-Sciences, Rydalmere, NSW, Australia).

A qualitative description of the distribution of lymphatics in the mouse uterus was made. Following VEGF-D overexpression, lymphatic (LVD) and blood (BVD) vessel density was determined in both the myometrium and endometrium (BVD only) at sites near (approximately 600 μm) and distal (lower uterine region) to the tumors. Sections were scanned at low magnification to determine areas of high vessel density. Blood and lymphatic vessel profiles were then counted from four fields of view using a ×20 objective lens and the mean vessel density established per mm^2^.

PCNA positive and negative vessels were counted in the same areas and a proliferation index calculated (percentage of vessel profiles containing ≥ 1 proliferating cells). The cross-sectional area of the five largest lymphatic vessel and blood vessel profiles located near and distal to the tumor (as described above) within the myometrium and endometrium were also measured.

### Statistics

Statistical analysis was performed using SPSS for Windows, version 14.0 (SPSS Inc., Chicago, IL, USA). A p-value less than 0.05 was considered significant. Measurements are presented as mean values ± SEM.

Two-way ANOVAs were used to compare the mean values for vessel density, proliferation and vessel size in relation to cell type (for VEGF-D expressing and control cells) and proximity to tumor (near and distal). If significant differences were found, Tukey HSD post hoc analysis was performed.

## Results

### Lymphatic vessels are largely restricted to the myometrium in mouse uterus

**Figure 1:**
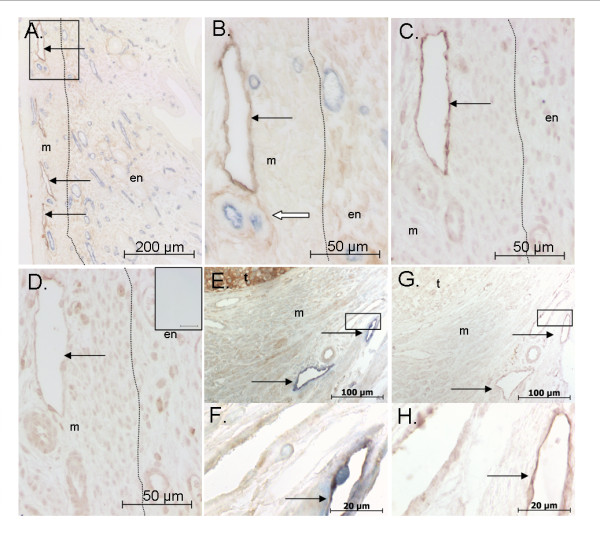
**Lymphatic vessels are largely restricted to the myometrium in mouse uterus**. (A) Representative photomicrograph of podoplanin (brown) and CD31 (blue) dual-immunostaining in mouse uterus highlighting the lymphatic and blood vessels, respectively. An area of a transverse section is shown. (B) Higher magnification of tissue within boxed area in A. Note the pale but distinct non-lymphatic podoplanin immunoreactivity in the connective tissue around the myometrial arteriole (white arrow). (C) Lyve-1 immunostaining is also useful for identifying uterine lymphatic vessels, as seen in this photomicrograph from a section serial to that in B. (D) VEGFR-3 immunostaining in a section serial to that in C (Inset: isotype-matched control for VEGFR-3). VEGFR-3 immunoreactivity is not useful as a marker of uterine lymphatics as varying levels of immunostaining are also observed in other uterine cellular components. (E, F) Representative photomicrographs of human VEGF-D (brown)/Lyve-1 (blue) and (G, H) VEGFR-3 immunostaining in NOD SCID mouse uterus inoculated with VEGF-D expressing 293EBNA cells. Note: F and H are higher magnification images of tissue within boxed area in E and G, respectively. Black arrows: lymphatic vessels. Dotted line delineates myometrium and endometrium. en: endometrium, m: myometrium, t: tumor.

To determine the distribution of lymphatic vessels in the mouse uterus, we initially examined Lyve-1 immunoreactivity in uterine sections from mice across the estrus cycle, during early pregnancy and in response to estrogen and progesterone treatment. Lyve-1-positive lymphatic vessels were almost exclusively observed in the connective tissue or stroma between the longitudinal and circular muscle layers of the myometrium (Figure 1). Lymphatic vessel profiles were also observed between the muscle bundles. [Potential differences in number and size of lymphatic vessels within the myometrium among different treatment groups have not been quantified in the current study.]

In contrast to the myometrium, Lyve-1 lymphatic vessel profiles were extremely rare within the endometrium. Lymphatic vessels were only observed on 17% (14 of 81 sections examined, 1-2 section per mouse) of the Lyve-1 immunostained sections examined (not specific to any particular treatment group). When lymphatic vessel profiles were observed, the sections usually contained only 1-2 profiles, which were nearly always situated directly adjacent to the endometrial/myometrial border.

In previous human studies using Lvye-1 immunostaining, lymphatic vessels were only detected within the myometrium, but not the endometrium [33,34]. To determine whether a population of Lyve-1 negative lymphatic vessels are also present in the mouse endometrium, we immunostained a subset of sections with antibodies against podoplanin and VEGFR-3.

As with Lyve-1, podoplanin-positive lymphatic vessels were observed predominately in the mouse myometrium (Figure 1A-B). Podoplanin immunoreactivity was also observed in the connective tissues surrounding the vascular smooth muscle cells of arterioles within the myometrium and sometimes in the basal regions of the endometrium. Additionally, non-vascular immunostaining was observed to varying degrees in the stromal fibroblasts in the basal region of the endometrium lying adjacent to the myometrium. No podoplanin immunostaining was observed in the sub-epithelial regions of the endometrium.

The VEGFR-3 antibody was not useful as a specific lymphatic vessel marker in mouse uterus (Figure 1D). Although the myometrial lymphatic vessels were VEGFR-3 positive, there was also varying amounts of VEGFR-3 immunoreactivity in other uterine components including the myometrium, stroma, epithelium and blood vessels. The immunostaining did not relate to a particular reproductive state. In other sections, individual cells within the endometrium labelled intensely with the VEGFR-3 antibody. Based on the above observations, the remaining analyses were conducted with the Lyve-1 antibody.

### VEGF-D is expressed by the mouse uterus

**Figure 2:**
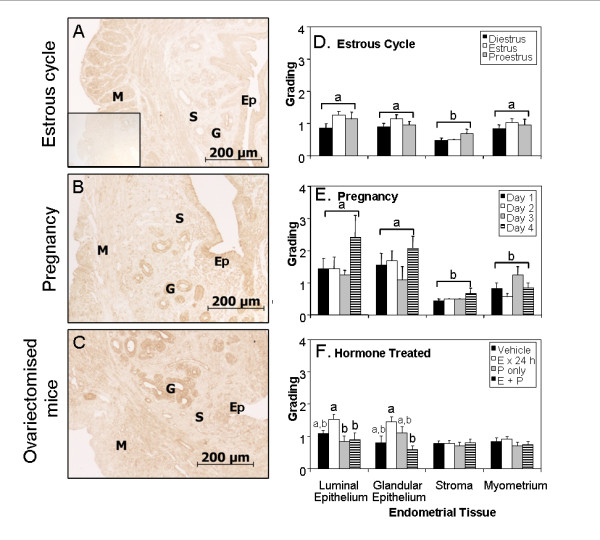
**VEGF-D immunostaining in the mouse uterus**. Representative photomicrographs (A-C) and semi-quantitative evaluation (D-F) of VEGF-D immunostaining in the mouse uterus. Representative photomicrographs from a normal cycling (estrous cycle) mouse (A), from a mouse in the early pregnancy (pre-implantation (B), and from an ovariectomised mouse (C). Inset in (A): isotyped-matched control. Ep: luminal epithelium, G: gland, M: myometrium, S: endometrial stroma. Semi-quantitative evaluation of VEGF-D (D-F) immunostaining in luminal epithelium, glandular epithelium, endometrial stroma and myometrium from the uterus of mice during the estrous cycle (D, n = 7-9), early pregnancy (E, days 1-4, n = 4-5) and in ovariectomised mice (F) following vehicle (n = 7), estradiol-17β (E × 24 h, n = 7), progesterone (P, n = 7), or progesterone following estradiol-17β priming (E + P, n = 8). Data are illustrated as means ± SEM. Groups that do not share a letter in common are significantly different (P < 0.05)

VEGF-D immunoreactivity was analysed in uteri collected from mice during the estrous cycle, in early pregnancy and following estrogen or progesterone treatment. VEGF-D immunostaining was observed in all uterine compartments (luminal epithelium, glandular epithelium, endometrial stroma and myometrium) in all sections examined, although VEGF-D immunostaining was generally low (Figure 2).

VEGF-D immunostaining varied significantly during the estrous cycle (F_(2,92) _= 9.6, p = 0.02); immunostaining was slightly but significantly more intense during estrus in comparison to diestrus (Figure 2D). VEGF-D immunostaining also varied significantly among the different uterine regions in mice collected at different stages of the estrous cycle (F_(3,92) _= 9.6, p < 0.001), with immunostaining significantly less intense in the stroma in comparison to the other regions examined.

VEGF-D immunostaining did not vary significantly across the first 4 days of pregnancy (F_(3,56) _= 2.2, p = 0.1, Figure 2E), although immunostaining did vary among the different uterine regions (VEGF-D: F_(3,56) _= 4.2, p < 0.001). VEGF-D immunostaining was significantly higher in the luminal and glandular epithelium in comparison to the stroma and myometrium.

VEGF-D immunostaining in ovariectomised mouse uterus varied significantly in response to exogenous hormone treatment (F_(3,116) _= 6.0, p = 0.001, Figure 2F). Immunostaining also varied among the different uterine compartments (VEGF-D: F_(3,116) _= 4.9, p = 0.003). Estradiol-17β treated animals had slightly, but significantly, higher VEGF-D immunostaining than mice treated with vehicle or progesterone (with or without estradiol-17β priming); this was particularly apparent in the luminal and glandular epithelium.

### Effects on the uterine vasculature in a mouse model of VEGF-D over-expression

To determine the response of the uterus to pathological over-expression of the VEGF-D polypeptide, we developed a mouse model in which the human 293EBNA line expressing VEGF-D was grown as a xenograft in the uterus of NOD/SCID mice. This is a model adapted from the skin tumor model previously published by some of us [25]. Tumors developed in all mice inoculated with either control 293EBNA cells (n = 9) or 293EBNA-VEGF-D cells (n = 9), either within or adjacent to (on mesometrium) the uterus. However, only uteri from mice that had tumors within the uterus (endometrium and/or myometrium) were analysed (uteri near tumors from mice receiving control 293EBNA cells: n = 9; uteri near tumors from mice receiving 293EBNA-VEGF-D cells: n = 7). Pieces of uteri lacking tumor were also collected from sites distal to the site of tumor cell injection (lower uterine horn). VEGF-D production by 293EBNA-VEGF-D cells within the mouse uterus was confirmed by immunohistochemistry. However, it was not possible to quantify VEGF-D production in the current model because of the variable number and distribution of cells within uterine tissues. Bioactivity of the VEGF-D produced by 293EBNA-VEGF-D cells has previously been confirmed by mitogenesis and miles assays [25].

### Over-expression of VEGF-D did not stimulate endometrial lymphangiogenesis

We used antibodies directed against Lyve-1 to examine the mouse uterine vasculature in response to over-expression of VEGF-D. As with the above observations, lymphatic vessels were almost exclusively located between the circular and longitudinal muscular layers of the uterine horn and occasionally adjacent to the endometrial-myometrial junction in NOD/SCID mice. No endometrial lymphatic vessels were observed in any of the samples analyzed from mice receiving either tumor cell type (n = 0/16). Uteri were also immunostained with an antibody directed against VEGFR-3. However, as noted in C57/CBA mice, immunostaining was not restricted to lymphatic vessels (contrast Figure 1E-F with Figure 1G-H). Although lymphatic vessels stained, low immunoreactivity was also noted in other uterine tissues including blood vessels. Therefore, Lyve-1 was used for analyses. VEGFR-3 immunoreactivity in tumor cells was negligible or absent (Figure 1G).

### VEGF-D over-expression caused proliferation and enlargement of existing myometrial lymphatic vessels

**Figure 3:**
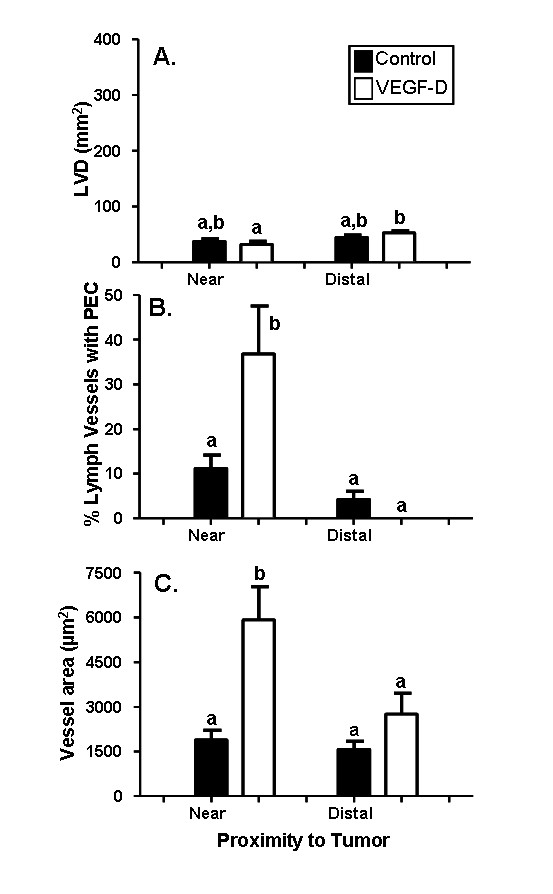
**Increased proliferation and surface area in myometrial lymphatic vessels in response to VEGF-D over-expression**. Uterine horns of NOD SCID mice were inoculated with control or VEGF-D expressing 293EBNA cells. The density of myometrial lymphatic vessels (A) did not vary between mice treated with VEGF-D expressing cells relative to control cells. However, the percentage of myometrial lymphatic vessels that contained proliferating cells (B) at sites adjacent to tumors was significantly increased in mice treated with VEGF-D cells, relative to control cells. Similarly, the surface area of myometrial lymphatic vessels (C) was significantly increased in sites adjacent to VEGF-D expressing tumors relative to control tumours. Black bars: control cells; White bars: VEGF-D cells. Data are illustrated as means ± SEM (n = 7-9). Groups that do not share a letter in common are significantly different (P < 0.05).

There was no significant difference in myometrial lymphatic density (LVD) between mice with 293EBNA-VEGF-D tumors versus control tumors (cell type: *F*(_1,32_) = 0.08, *p *= 0.78). However, LVD was significantly reduced near 293EBNA-VEGF-D tumors versus sites distal from 293EBNA-VEGF-D tumors (p = 0.04). This reduction in LVD was not observed in mice with control tumors (p = 0.68; Figure 3A).

**Figure 4:**
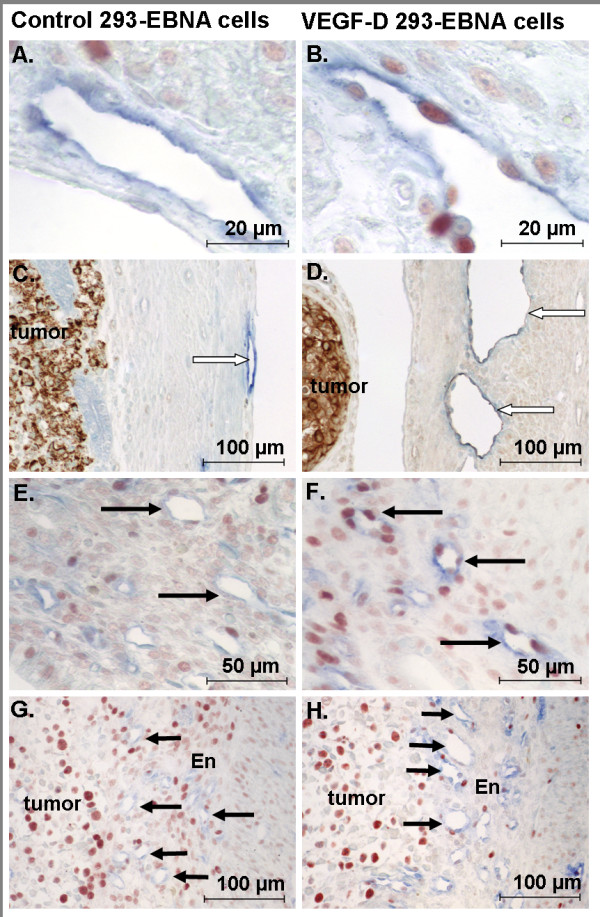
**Vasculature in uteri from NOD SCID mice inoculated with control or VEGF-2-expressing 293EBNA cells**. Representative photomicrographs of uterine horns from NOD SCID mice inoculated with control (A, C, E, G) or VEGF-D (B, D, F, H) expressing 293EBNA cells. (A, B) Myometrial lymphatic endothelial cell proliferation was increased in mice treated with VEGF-D expressing cells relative to those treated with control cells. Red: PCNA positive proliferating cells, Blue: Lyve-1 positive lymphatic vessel profiles. (C, D) Myometrial lymphatic vessels (white arrows). Note increased size of vessels in uterus exposed to VEGF-D. Brown: tumor cells (C: human mitochondrial positive cells, D: human VEGF-D positive cells). (E, F) There were also increased numbers of proliferating endometrial blood endothelial cells (adjacent to tumors) in mice treated with VEGF-D expressing cells relative to control cells. Red: PCNA positive proliferating cells, blue: CD31 positive blood vessel profiles. Black arrows: blood vessels. (G, H) Endometrial blood vessels (black arrows, note increased vessel size in uterus exposed to VEGF-D). Red: PCNA positive proliferating cells, blue: CD31 positive blood vessel profiles.

In contrast to LVD, both the amount of endothelial cell proliferation (*F*(_1,32_) = 4.32, *p *= 0.047, Figure 3B, 4A and 4B) and the vessel cross sectional areas (*F*(_1,32_) = 17.4, *p *< 0.001, Figure 3C, 4C and 4D) were significantly affected by VEGF-D over expression. Post hoc analysis confirmed that both variables were significantly increased near the 293EBNA-VEGF-D tumors when compared to that near the control tumors and distal to both tumor types. The increase in endothelial cell proliferation may contribute to the increase in lymphatic vessel size.

### VEGF-D over-expression stimulated enlargement and proliferation of endometrial, but not myometrial blood vessels

**Figure 5:**
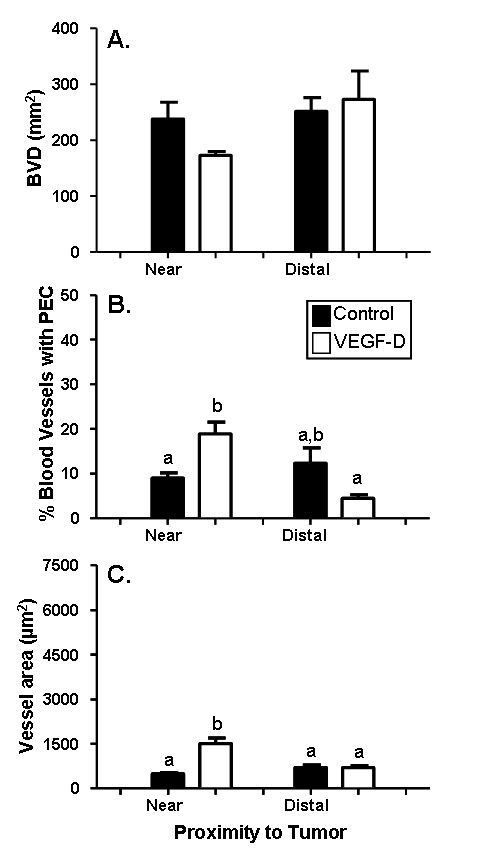
**Increased proliferation and surface area in endometrial blood vessels in response to VEGF-D over-expression**. Uterine horns of NOD SCID mice were inoculated with control or VEGF-D expressing 293EBNA cells. The density of endometrial blood vessels (A) did not vary between mice treated with VEGF-expressing cells relative to control cells. However, the percentage of endometrial blood vessels that contained proliferating cells (B) at sites adjacent to tumors was significantly increased in mice treated with VEGF-D cells, relative to control cells. Similarly, the surface area of endometrial blood vessels (C) was significantly increased in sites adjacent to VEGF-D expressing tumors relative to control tumours. Black bars: control cells; White bars: VEGF-D cells. Data are illustrated as means ± SEM (n = 7-9). Groups that do not share a letter in common are significantly different (P < 0.05).

The presence of 293EBNA tumor cells caused a similar response in the endometrial blood vessels as was observed in the myometrial lymphatic vessels described above. There was no significant difference between endometrial BVD at sites either near or distal to 293EBNA-VEGF-D compared to control tumors (Figure 5A). VEGF-D over-expression caused significant changes in endometrial blood vessel proliferation (*F*(_1,32_) = 5.36, *p *= 0.028, Figure 4E, F and 5B) and the cross sectional area of vessels (*F*(_1,32_) = 23.77, *p *< 0.001, Figure 4G, H and 5C). The percentage of proliferating blood vessels and the area of blood vessels near 293EBNA-VEGF-D tumors was significantly increased compared to near control tumors and distal to the both tumor types.

**Figure 6:**
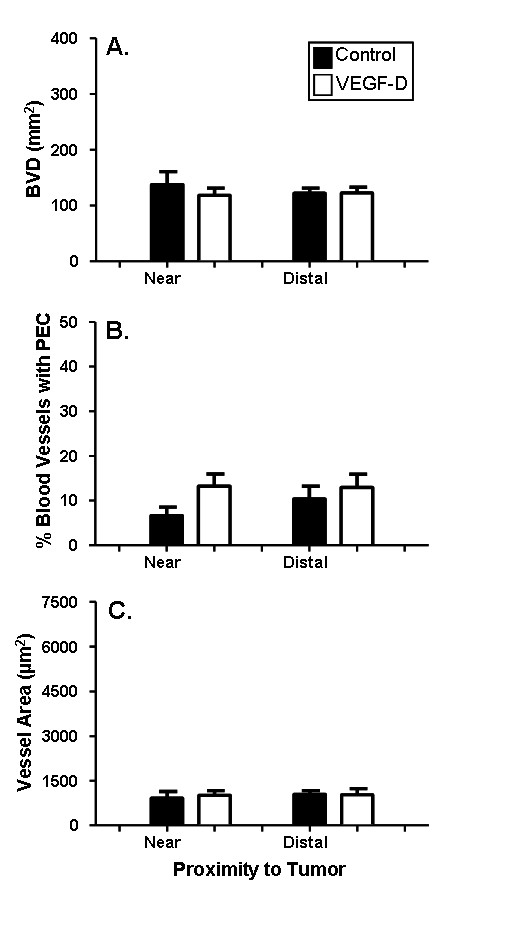
**No significant change in myometrial blood vessels in response to VEGF-D over-expression**. Uterine horns of NOD SCID mice were inoculated with control or VEGF-D expressing 293EBNA cells. The density of myometrial blood vessels (C) did not vary between mice treated with VEGF-expressing cells relative to control cells. There was also no significant difference in the percentage of myometrial blood vessels (B) that contained proliferating cells or the surface area of myometrial blood vessels (C) in mice with VEGF-D expressing tumors relative to control tumours. Black bars: control cells; White bars: VEGF-D cells. Data are illustrated as means ± SEM (n = 7-9).

The changes observed in the myometrial lymphatics and the endometrial blood vessels were not observed in the myometrial blood vessels. There was no change in myometrial BVD (Figure 6A), the percentage of myometrial blood vessels containing proliferating endothelial cells (Figure 6B), or the surface area of myometrial blood vessels (Figure 6C).

### VEGF-D tumor cells were observed in myometrial lymphatic vessels

**Figure 7:**
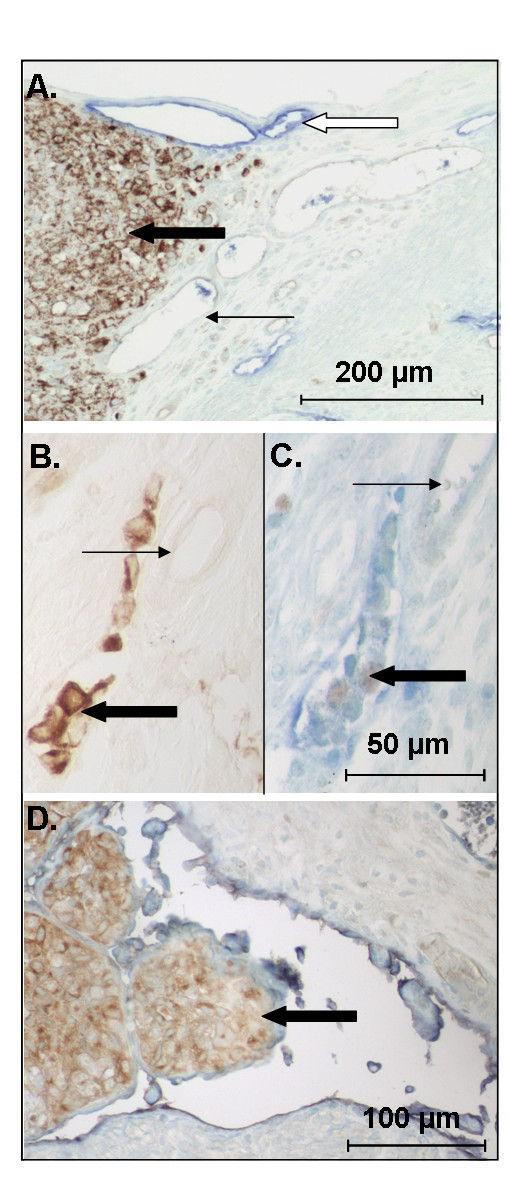
**VEGF-D expressing 293EBNA cells invade uterine lymphatic vessels**. Uterine horns of NOD SCID mice were inoculated with control or VEGF-D expressing 293EBNA cells. (A) Control tumor cells (brown) did not invade uterine blood or lymphatic (blue) vessels; however, (B-D) examples of myometrial lymphatic vessels containing tumor cells were observed in all mice treated with VEGF-D expressing cells. Note the tumor cells in a lymphatic vessel adjacent to unaffected blood vessels in B and C. Note the large tumor emboli in the distorted lymphatic vessel in D. (A and B: brown: tumor cells; C and D; blue: lymphatic vessels, brown: PCNA positive proliferating cells). Heavy black arrow: tumor cells. Fine black arrows: blood vessel. White arrow: lymphatic vessel.

A common feature of endometrial cancer is vascular space invasion by tumor cells. In the current study, none of the control tumor cells had invaded the myometrial lymphatic vessels (n = 0/9), while examples of myometrial lymphatic vessels containing invading cells or tumor emboli were observed in all uteri receiving 293EBNA-VEGF-D cells (n = 7/7) (Figure 7). Similar invasion in blood vessels was not observed.

## Discussion

We have shown that lymphatic vessels positive for the key lymphatic endothelial cell markers Lyve-1, podoplanin and VEGFR-3 are largely restricted to the connective tissue between the longitudinal and circular layers of the myometrium in the mouse uterus; only very few lymphatic vessels were present within the mouse endometrium and when observed were nearly always situated adjacent to the myometrial-endometrial border. We have also demonstrated that the presence of tumor cells over-expressing human VEGF-D has differential effects on the uterine vasculature in a mouse model of endometrial cancer. Unexpectedly, 293EBNA cells expressing human VEGF-D did not stimulate growth of new (lyve-1 positive) lymphatic vessels into the mouse endometrium, although they did stimulate changes in the pre-existing lymphatic vessels of the myometrium and blood vessels of the endometrium; no significant changes were observed in the myometrial blood vessels.

Until relatively recently, it was necessary to conduct detailed histological, ultrastructural or functional studies to describe the distribution of lymphatic vessels. The available techniques all have associated limitations and it is not always possible to accurately differentiate lymphatic vessels from blood vessels or tissue spaces. Specific lymphatic endothelial cell markers (including Lyve-1, podoplanin and VEGFR-3) are now available enabling detailed studies of lymphatic growth and function. In the current study, we have used three markers (Lyve-1, podoplanin, VEGFR-3) to examine the distribution of lymphatics in the mouse uterus. Our observation that the majority of lymphatic vessel profiles positive for Lyve-1, podoplanin and VEGFR-3 are situated within the myometrium is consistent with several earlier detailed histological and functional studies in mice, rats, rabbits and sheep [35-40]. In these studies, a well developed lymphatic vasculature was observed in the connective tissue separating the two myometrial muscle regions with smaller lymphatic capillaries extending into the longitudinal and circular muscle layers. Most of these studies report few lymphatic vessels in the endometrium. However, some studies report contrary results suggesting the presence of extensive endometrial lymphatic capillaries in mice and rats [35,40]. There have also been conflicting results in studies using lymphatic-specific endothelial cell markers. While Donoghue et al. [4] describe a distinct population of lymphatic vessels in the human endometrium based on podoplanin (D2-40) immunohistochemistry, other studies using Lyve-1 report an absence of lymphatics in the endometrium [33,34]. It is apparent that caution is still required when examining lymphatic vessels within uterine tissues, even when lymphatic endothelial cell markers are used. In addition, it will be important for future researchers to investigate the functional differences in uterine vessel populations exhibiting differential lymphatic-endothelial specific marker expression.

In this study, we used a VEGF-D over-expressing tumor cell line as a model of VEGF-D over-expression in endometrial cancer. We chose to use the 293EBNA-VEGF-D cells as they had previously been shown to induce lymphangiogenesis and angiogenesis in NOD/SCID mice [25]. In addition, the original 293EBNA cells do not express detectable levels of VEGF-A, VEGF-C or VEGF-D, reducing any confounding effects of other key VEGF family members on our analyses. The 293EBNA-VEGF-D cells express full-length VEGF-D (53 kD), some of which is proteolytically processed to intermediate and fully processed forms with higher affinities for its cognate receptors VEGFR-2 and VEGFR-3 on endothelium. The VEGF-D produced by these cells was previously shown to induce tumor angiogenesis and lymphangiogenesis and to promote tumor metastasis to local lymph nodes when cells were injected subcutaneously [25]. VEGF-D has also been shown to induce lymphangiogenesis in several other models following cell-mediated over-expression [25], adenoviral delivery [41,42] and transgenic expression [43].

Although the presence of VEGF-D-expressing 293EBNA tumor cells did not induce endometrial lymphangiogenesis, they did cause differential effects on the existing uterine vasculature. Increased proliferation and vessel size was observed in the myometrial lymphatic and endometrial blood vessels. Similar vessel enlargement has been observed in rabbit and mouse hind limb [24,42], rat cremaster muscle [41], pig heart [44] and mouse skin [43] in response to VEGF-D. In contrast, no significant changes were noted in the myometrial blood vessels. This differential response to VEGF-D might be due to differences in the local tissue microenvironment or the particular response of these tissue-specific vessels. Adenoviral VEGF-D (AdvVEGF-D) over-expression in mouse skin induces increased angiogenesis and lymphangiogenesis with vessel enlargement [41,42], while in the lung AdvVEGF-D induces lymphangiogenesis without angiogenesis [45]. Alternatively, the differential responses may reflect the location of blood or lymph vessels on the vascular tree. The small endometrial capillaries, which have less mural cell (pericytes and vascular smooth muscle cells) support than the more stable blood arterioles present in the connective tissue of the myometrium [1], are likely to be more responsive to angiogenic promoters. However, this latter hypothesis does not explain the lack of response in small capillaries present within the muscle itself.

It has been proposed that VEGF-D is crucial to carcinoma-associated lymphangiogenesis, as well as the process of metastasis [12,25]. In the current study, 293-EBNA-VEGF-D cells or tumor emboli were observed within myometrial lymphatic vessels. In contrast, no invasion of lymphatic vessels was observed in mice receiving control 293EBNA cells. The presence of emboli in the vasculature is a prognostic factor in several cancers, including endometrial cancer (see [8,46,47] and references therein). Invasion of the vasculature is also thought to be an early step in the metastatic process. The mechanism by which VEGF-D might induce vascular invasion and ultimately metastasis is not known. Although VEGF-D over-expression caused enlargement and proliferation of uterine lymphatic vessels, these effects in themselves are not sufficient to explain the observed migration of tumor cells into uterine vessels. Future research will be needed to determine whether vessel invasion reflects characteristics of the endometrial VEGF-D expressing tumor cells or because of changes induced in the vessels affected.

In addition to our focus on endometrial cancer, the observed effects of VEGF-D over-expression on uterine vasculature raises questions about the function of this growth factor in normal uterine remodelling. In this study, no new growth of lymphatic vessels into the endometrium was induced. A possible explanation for this lack of growth is that lymphangiogenesis is actively inhibited within the endometrium. Such inhibition has been observed in the cornea, which must remain avascular to maintain the transparency required for vision. It has been shown that constitutive expression of VEGFR-3 on the corneal epithelium acts as a sink for VEGFR-3 ligands, thereby suppressing vascular growth into the cornea [48]. In addition, a recent study has identified a soluble splice variant of VEGFR-2 in mice that inhibits lymphangiogenesis by blocking VEGF-C. This isoform specifically blocks injury-induced lymphangiogenesis, but not hemangiogenesis, in the mouse cornea [49]. Several studies have described varying amounts of non-lymphatic VEGFR-3 immunostaining within the endometrial epithelium and stroma [50-52], however, the activity of this non-lymphatic VEGFR-3 remains to be elucidated. Whether a soluble isoform of VEGFR-2 is expressed in the uterus has not yet been investigated.

In the current study, mouse uterus was treated with human VEGF-D which interacts with both mouse and human VEGFR-2 and VEGFR-3. It should be noted, however, that mouse VEGF-D does not bind with VEGFR-2 [21] and it is unlikely that similar blood vessel responses would have been observed if tumour cells had been transfected with mouse VEGF-D. However, as human VEGF-D does bind with human VEGFR-2, future studies need to address the functional role of VEGF-D in human endometrium with emphasis on the differential effects on different vascular components within the tissue.

Future studies should also consider the role of VEGF-C in endometrial vascular remodeling. As with VEGF-D, VEGF-C can interact with both VEGFR-2 and VEGFR-3, and has been shown to induce both angiogenesis and lymphangiogenesis in various tissue and pathology models [53-55]. Depending on the nature of the ligand and the presence of co-receptors, the VEGF receptors can form both homo and heterodimers with subsequent auto-phosphorylation of the receptor complex and downstream signaling; the associated variation in signaling pathways associated with homo versus heterodimers has yet to be elucidated (reviewed in [56,57]). The specific interactions of VEGF-D with VEGF receptors has not been examined in the current model, but future research in this area will highlight the mechanisms by which VEGF-D acts to promote lymphangiogenesis and metastasis in endometrial cancer. While this study has focused on the effects of VEGF-D overexpression on vessel morphology and endothelial cell proliferation, future research will need to consider the other processes influenced by VEGF receptor activation (cell survival, cell migration, vessel permeability). Modification of any of these processes would also likely contribute to VEGF-D's role in endometrial cancer progression.

The NOD SCID mice used in the current study have various defects of immune function including decreased numbers of circulating T lymphocytes, B lymphocytes and natural killer cells. This means that any effects of VEGF-D overexpression on the uterine lymphocyte population could not be investigated in this model. The profiles of VEGF-D and VEGFR-3 expression may also vary in the uterus of these mice relative to those in the C57/CBA mice used for the initial analysis. Despite the above, our study has shown a clear effect of VEGF-D over-expression on uterine vasculature.

## Conclusions

In conclusion, we have shown that lymphatic vessels positive for key lymphatic specific markers Lyve-1, podoplanin and VEGFR-3 are largely restricted to the myometrium in the mouse uterus. We have also demonstrated in a model of endometrial cancer, that VEGF-D over-expressing tumor cells do not stimulate growth of lymphatic vessels into the endometrium. This may be due to a lack of appropriate small lymphatic vessels/capillaries in the uterus on which VEGF-D can act. Alternatively, an inhibitor(s) preventing lymphangiogenesis may be present within the endometrium. Although no endometrial lymphangiogenesis was observed, VEGF-D over-expression did stimulate proliferation and an increase in the size of existing uterine vasculature. VEGF-D expressing tumor cells were also observed within myometrial lymphatic vessels; whether this vascular invasion reflects characteristics of the VEGF-D tumor cells or induced-changes in the uterine vasculature remain to be elucidated.

The results obtained in the current study show that VEGF-D over-expression has differential effects on the uterine vasculature. These effects may facilitate VEGF-D's ability to promote endometrial cancer metastasis and disease progression. The results also highlight the utility of the current model as a tool to investigate endometrial carcinoma-associated VEGF-D expression and activity.

## Competing interests

JFD, FLL and LMC have nothing to declare. PAWR, JEG, MGA and SAS received grant support from NHMRC as outlined below. MGA and SAS are paid consultants on the Advisory Board of Vegenics Ltd. They have received royalties from Vegenics Ltd and own stock in Circadian Technologies (owns Vegenics). MGA and SAS are inventors on the VEGF-D patent. MGA has acted as an expert witness in patent hearings.

## Authors' contributions

JEG was involved in the design of the studies, participated in the tissue analysis, undertook data analysis and drafted the manuscript. JFD was involved in the design of, and conducted, the VEGF-D over-expression studies and participated in tissue and data analysis and drafting the manuscript. FLL and LMC were involved in the development and undertaking of immunohistochemistry. MGA and SAS participated in the experimental design and provided tumor cell lines and associated technical assistance and advice. PAWR designed the experiment and contributed to data interpretation. All authors read and approved the final manuscript.
